# Supplementary material for: The effects of skill-based health education—A randomised-controlled intervention in primary schools in rural Bangladesh
Source: PLoS One. 2025 Jul 11;20(7):e0327325. doi: 10.1371/journal.pone.0327325 (PMC12250694; doi:10.1371/journal.pone.0327325)
Supplement: S1 Zip — S1 Fig. Project School Map in Jhenaidah, Bangladesh. S1 Table. Endline (non-DID) estimation of family-wise mean-standardised effect in average effect size on nine outcome families adjusting for baseline covariates (all children). S2 Table. DID estimation of family-wise mean-standardised effect in average effect size on nine outcome families with additional covariates (all children). S3 Table. DID estimation of family-wise mean-standardised cross-cutting HESP-treatment effect in average effect size on five selected outcome families with additional covariates (all children). S4 Table. HE-treatment effects on single outcomes (selected outcomes) (all children; children in both surveys) S1 File. Study Protocol. S1 Checklist. CONSORT Checklist. (ZIP) [file pone.0327325.s001.zip › supplements/S1 Table.pdf]

S1 Table. Endline (non-DID) estimation of family-wise mean-standardised effect in average effect size on nine outcome families adjusting for baseline covariates (all children)

	Primary Outcomes								
	(P1) handwashing			(P2) dentalcare			(P3) overall hygiene		
	AES-coefficient [95%CI] [p-value]			AES-coefficient [95%CI] [p-value]			AES-coefficient [95%CI] [p-value]		
HE-treatment	0.18***	0.17***	0.17***	0.16***	0.15**	0.16***	0.15***	0.15***	0.15***
	[0.11,0.24]	[0.10,0.24]	[0.11,0.24]	[0.07,0.25]	[0.06,0.25]	[0.07,0.25]	[0.10,0.21]	[0.09,0.21]	[0.09,0.21]
	[0.000]	[0.000]	[0.000]	[0.001]	[0.001]	[0.001]	[0.000]	[0.000]	[0.000]
school type	-0.03	-0.05	-0.04	-0.07	-0.08	-0.06	-0.03	-0.06+	-0.04
	[-0.09,0.03]	[-0.12,0.02]	[-0.10,0.03]	[-0.16,0.02]	[-0.17,0.02]	[-0.15,0.03]	[-0.09,0.03]	[-0.12,0.01]	[-0.10,0.02]
	[0.347]	[0.146]	[0.277]	[0.154]	[0.104]	[0.165]	[0.283]	[0.081]	[0.160]
sex	0.09***	0.10***	0.10***	0.02	0.02	0.02	0.06***	0.07***	0.07***
	[0.06,0.12]	[0.07,0.13]	[0.07,0.13]	[-0.02,0.05]	[-0.02,0.06]	[-0.02,0.06]	[0.04,0.08]	[0.05,0.09]	[0.05,0.09]
	[0.000]	[0.000]	[0.000]	[0.427]	[0.295]	[0.281]	[0.000]	[0.000]	[0.000]
child age		0.02***			0.02**			0.03***	
		[0.01,0.03]			[0.00,0.03]			[0.02,0.04]	
		[0.000]			[0.008]			[0.000]	
wealth index			0.10***			0.10***			0.10***
			[0.08,0.12]			[0.08,0.13]			[0.08,0.11]
			[0.000]			[0.000]			[0.000]
N	6111	6113	6104	6113	6113	6104	6098	6105	6096

	(P4) clean hands			(P5) nutrition			(P6) knowledge		
	AES-coefficient [95%CI] [p-value]			AES-coefficient [95%CI] [p-value]			AES-coefficient [95%CI] [p-value]		
HE-treatment	0.02	0.03	0.03	0.01	0.01	0.01	0.34***	0.34***	0.34***
	[-0.06,0.11]	[-0.06,0.11]	[-0.05,0.12]	[-0.03,0.04]	[-0.03,0.05]	[-0.03,0.05]	[0.28,0.41]	[0.27,0.41]	[0.28,0.41]

	[0.577]	[0.546]	[0.471]	[0.671]	[0.542]	[0.536]	[0.000]	[0.000]	[0.000]
school type	-0.06	-0.06	-0.05	0.03+	0.03	0.04*	0.03	0.02	0.02
	[-0.14,0.03]	[-0.15,0.03]	[-0.14,0.04]	[-0.00,0.07]	[-0.01,0.07]	[0.00,0.07]	[-0.04,0.10]	[-0.05,0.09]	[-0.04,0.09]
	[0.188]	[0.173]	[0.255]	[0.073]	[0.116]	[0.043]	[0.397]	[0.653]	[0.493]
sex	0.10***	0.12***	0.12***	0	0.01	0.01	-0.01	-0.01	-0.01
	[0.06,0.15]	[0.08,0.16]	[0.08,0.17]	[-0.03,0.03]	[-0.02,0.04]	[-0.02,0.04]	[-0.05,0.03]	[-0.05,0.03]	[-0.05,0.03]
	[0.000]	[0.000]	[0.000]	[0.882]	[0.647]	[0.569]	[0.594]	[0.562]	[0.646]
child age		0.05***			0.01			0.05***	
		[0.04,0.07]			[-0.00,0.02]			[0.04,0.06]	
		[0.000]			[0.125]			[0.000]	
wealth index			0.09***			0.06***			0.07***
			[0.06,0.12]			[0.04,0.07]			[0.05,0.09]
			[0.000]			[0.000]			[0.000]
N	6108	6112	6103	6113	6113	6104	6113	6113	6104

Secondary Outcomes

	(I1) cold-related symptoms			(I2) other illness			(I3) anthropometry		
	AES-coefficient [95%CI] [p-value]			AES-coefficient [95%CI] [p-value]			AES-coefficient [95%CI] [p-value]		
HE-treatment	-0.02	-0.02	-0.02	-0.02	-0.02	-0.02	-0.02	0.01	-0.021
	[-0.06,0.02]	[-0.06,0.02]	[-0.06,0.02]	[-0.06,0.03]	[-0.06,0.03]	[-0.06,0.03]	[-0.04,0.01]	[-0.05,0.08]	[-0.06,0.02]
	[0.284]	[0.361]	[0.347]	[0.498]	[0.500]	[0.477]	[0.211]	[0.679]	[0.285]
school type	-0.01	-0.01	-0.02	0.01	0.01	0	-0.01	-0.01	-0.009
	[-0.06,0.03]	[-0.06,0.03]	[-0.06,0.02]	[-0.04,0.05]	[-0.04,0.05]	[-0.04,0.05]	[-0.03,0.02]	[-0.07,0.06]	[-0.07,0.05]
	[0.482]	[0.494]	[0.394]	[0.699]	[0.750]	[0.859]	[0.599]	[0.822]	[0.768]
sex	0	0	0	0.08***	0.09***	0.09***	0.03*	-0.02	-0.048**
	[-0.03,0.03]	[-0.03,0.03]	[-0.03,0.03]	[0.05,0.11]	[0.06,0.12]	[0.06,0.12]	[0.00,0.05]	[-0.06,0.03]	[-0.08,-0.01]

	[0.940]	[0.977]	[0.967]	[0.000]	[0.000]	[0.000]	[0.032]	[0.399]	[0.011]
child age		-0.01+			-0.02**			-0.03***	
		[-0.02,0.00]			[-0.03,-0.01]			[-0.05,-0.01]	
		[0.067]			[0.002]			[0.000]	
wealth index			-0.03**			-0.03**			
			[-0.04,-0.01]			[-0.04,-0.01]			
			[0.008]			[0.003]			
N	6113	6113	6104	6104	6113	6104	6094	6102	16130

Notes: Each column represents a separate regression on a family of endline outcomes applying seemingly unrelated regressions (SUR), estimated by a feasible generalised least squares (FGLS) estimator with cluster-robust standard errors (CRSE). AES-coefficient is the mean-standardised average effect size. Analysis is conducted for all children, controlling for school type, child sex, baseline outcomes (estimates omitted) and additional covariates, namely, *child age* and *wealth index*. *Wealth index* is created through iterated principal factor, reflecting house structure materials, roof materials, number of rooms, latrine structure and materials, possession of electronic appliances, mobile phones and bikes. Both endline and baseline outcomes are normalised by subtracting the control group baseline mean values and divided by the control group baseline standard deviation as in Kling et al. (2007) and Clingingsmith et al. (2009). Each indicator family includes the following variables: (P1) *handwashing practice*: handwashing frequency in each occasion (before eating, after defecation, after playing), used substances (soap, ash, mud and/or water only), washing with soap in each occasion, wash with running water, correct washing procedure; (P2) *dentalcare practice*: frequency of dentalcare, frequency of using brush/branch, type of materials used; (P3) *overall hygiene practice*: shoes/footwear wearing at school (frequency), shoes/footwear wearing at home (frequency in latrine and in courtyard), + P1 & P2; (P4) *clean hand*s: clean hands by observation, trimmed nails, clean nails; (P5) *nutrition practice*: breakfast habit, breakfast taken in 3 days, food taken in 3 days, ordered by the richness of nutrition score (none; carbohydrate (and fat); carbohydrate and vitamins; vegetable/animal protein and vitamins; vegetable/animal protein and carbohydrate; vegetable protein, animal protein and carbohydrate; protein, carbohydrate and vitamins); (P6) *health/hygiene knowledge*: handwashing procedure, breakfast significance; (I1) *cold-related symptoms*: symptoms at present and in the past two-weeks of cough, breathing difficulty, sore throat, fever, running nose, congested nose; (I2) *other illness*: diarrhoea, stomachache, skin disease, fatigue, dizziness, appetite loss in the past two-weeks; (I3) *anthropometry*: height-, weight-, BMI-z-score. Significance level: + p<0.1, * p<0.05, ** p<0.01, ***p<0.001; 95% confidence intervals and p-value in brackets.
