# Supplementary material for: The effects of skill-based health education—A randomised-controlled intervention in primary schools in rural Bangladesh
Source: PLoS One. 2025 Jul 11;20(7):e0327325. doi: 10.1371/journal.pone.0327325 (PMC12250694; doi:10.1371/journal.pone.0327325)
Supplement: S1 Zip — S1 Fig. Project School Map in Jhenaidah, Bangladesh. S1 Table. Endline (non-DID) estimation of family-wise mean-standardised effect in average effect size on nine outcome families adjusting for baseline covariates (all children). S2 Table. DID estimation of family-wise mean-standardised effect in average effect size on nine outcome families with additional covariates (all children). S3 Table. DID estimation of family-wise mean-standardised cross-cutting HESP-treatment effect in average effect size on five selected outcome families with additional covariates (all children). S4 Table. HE-treatment effects on single outcomes (selected outcomes) (all children; children in both surveys) S1 File. Study Protocol. S1 Checklist. CONSORT Checklist. (ZIP) [file pone.0327325.s001.zip › supplements/S1 File study protocol.pdf]

Study Protocol

A Randomized-Controlled Intervention and Evaluation of an Innovative School Health and Nutrition Education (ISHNE) project for Primary Schools in Rural Bangladesh

A proposed study in partnership with Meiji Gakuin University in Tokyo, Save the Children USA, the Ministry of Primary and Mass Education and the Director General of Health Services Bangladesh.

June 2011

Meiji Gakuin University Contact Information:
Principal Investigator: Makiko Omura
Associate Professor
Meiji Gakuin University
1-2-37 Shirokanedai, Minato-ku, Tokyo
108-8636 JAPAN
Tel: +81-3-5421-5340
Fax: +81-3-5421-5207
E-mail: makiko@eco.meijigakuin.ac.jp

Country Office Contact Information:

Country Director, Bangladesh
Save the Children Federation, Inc.
House 1 (A) Road 91, Gulshan 2
Dhaka
Tel: (880-2) 911-5291
Fax: (880-2) 811-3019
E-mail: @savechildren.org

This project forms a part of the “Grant-in-Aid for Scientific Research (B) (23402033),” entitled *A Field Experiment to Evaluate Health Education Methods: For Sustainable Health Improvement of Impoverished Children in Developing Countries*, funded by the Japan Society for the Promotion of Science (JSPS).

Title: A Randomized-Controlled Intervention and Evaluation of an Innovative School Health and Nutrition Education (ISHNE) project for Primary Schools in Rural Bangladesh

This study aims to evaluate the impact and cost-effectiveness of innovative school health and nutrition education (ISHNE) interventions at primary schools on behavior change, health outcomes and school attendance. The ISHNE consists of:

(1) Weekly health education sessions using **participatory learning methods** for **skill-based health education** (SBHE)
(2) Use of primary school **para-teachers** instead of teachers as the facilitators of the health education sessions.
(3) Use of **mobile phone technology**[1] to assist pedagogy and learning, and to motivate students and teachers
(4) Distribution of bars of soap in order to identify the impact of goods provision vs. awareness raising

Background

Global Context

Education, as envisioned by the Convention on the Rights of the Child, is a basic human right; and given its high returns on human capital, a key factor to the development of societies. With this understanding international agencies and governments launched a global initiative to ensure Education for All at the World Education Forum in 2000. Poor health status of school-age children (SAC), aged 5-14 years, however presents a key barrier for ensuring access to quality education. Between 25 and 35 percent of SAC in the world are infected with parasitic worms, 48 to 56 percent are stunted, 53 percent suffer from iron deficiency anemia, 5 percent remain affected by iodine deficiency and 7 percent by vitamin A deficiency. In many cases, children, teachers and parents don't even know they have a health problem, and their under-nutrition, fatigue and inability to concentrate is considered normal. But these mild chronic infections may have a substantial effect on their emotional and psychological well-being, their ability to learn and attend school, and make healthy life choices.[2]. School-based health services such as mass deworming and micronutrient supplementation have proven to be effective and extremely successful in reducing disease morbidity and malnutrition. However, their effect can be short-term – children are still at risk of being re-infected with worms due to poor hygienic practices and having micronutrient deficiencies due to a poor diet. The fundamental factor to achieving sustainable health improvements in the long-term is the development of positive attitudes and behaviors, as many of these illnesses can be mitigated by adopting healthy behaviors such as simple hand-washing with soap.[3] [4]

Globally, skills -based health education (SBHE) has been found to be a highly effective means to promoting healthy behaviors in children. Rigorous research, particularly from high-income countries have found SBHE to improve skills in relation to health concerns, reinforce positive behaviors, and to reduce health problems, while also improving students' achievement scores[5]. SBHE focuses on the development of knowledge, attitudes, and *especially skills*, in children, and an essential element to achieving this is the use of a variety of participatory learning methods (such as class discussions, role-play, games, small group work and action research) and the emphasis on learning outcomes and life-skills- such as problem-solving, negotiation and communication. Save the Children (SC), has implemented many skills-based health education projects, both as in-class stand alone as well as out-of-class peer-led sessions.

SBHE projects, however, are considered expensive, labor intensive and requiring long-term involvement, which projects find unsustainable to replicate to scale. Due to the difficulties in measuring change, many project evaluations have also unfortunately found health education to be ineffective, especially in comparison to drug/ supplement provision[6]. A key challenge under *program conditions* has been *motivating* teachers to conduct health education, and to do it effectively, and encouraging children to change their behaviors. Teachers and children in resource poor settings often do not have the relevant *information* or *skills* to adopt healthy behaviors, and the *environment* in which they learn is not conducive to these behaviors either. Often children perform poorly academically due to the disconnection between education content and their experience. If content were made more relevant and reinforced learning outcomes and skills, both teachers and students may be more motivated to teach and learn. If SBHE projects were more innovative in making content interesting and engaging, without much effort from teachers or large investments needed for replication at scale, implementation would be more successful.

Situation in Bangladesh

In Bangladesh, despite efforts by the government and international community in providing anti-helminth drugs, supplements and sanitary infrastructures to schools, children are still suffering from preventable diseases. According to latest statistics, the major illnesses affecting SAC are: diarrhea, micronutrient deficiencies, malnutrition, intestinal helminthes, pneumonia, assault, typhoid, and scabies.[7,8]

In order to address these concerns development partners in Bangladesh, including UNICEF, World Bank, WFP, GTZ, and SC have been working with the government to develop a framework for incorporating SHN in to

Bangladesh's national education strategic plan (PROG3) currently being drafted for the 2011-2016 period. A proposed intervention to the Ministry of Primary and Mass Education (MoPME) and the Ministry of Health and Family Welfare (MoHFW) has been to hold school health days six-monthly, which coincide with national deworming days, where health services are provided to Bangladesh's 20 million SAC.

However, health services alone not being sufficient, the MoPME is introducing a health education curriculum for primary schools called Better Health Better Education (BHBE). This new curriculum will be used as a supplement to textbooks in classrooms, and where possible practical demonstrations will also take place. Teachers (one per school) from 7 out of Bangladesh's 64 districts have been trained in BHBE, and they are currently in the process of incorporating these messages in their lesson plans. Despite the introduction of BHBE, following challenges with the present system are identified: **a)** textbooks do not contain certain important health information till later in the academic life of a child (In Bangladesh where the drop out rate is nearly 50% by the end of grade 3, messages on nutrition only begin in grade 4 and are missed by half of the school-going population); **b**) opportunities for repeating and reinforcing messages are limited since the information is contained in a specific chapter in a textbook, and the health content along with the chapter may be covered on average only once over the school year; **c)** health content competes with other information in the subject and chapter within the textbook, which also needs to be discussed by the class in the limited time allotted; **d**) teachers already overburdened with covering many topics on the curriculum, resort to traditional rote learning methods, as opposed to a skills-based education; and **e**) occasional practical demonstrations are not sufficient in reinforcing behaviors.

In order to address these challenges, and general difficulties with SBHE mentioned earlier, SC proposes an innovative approach to the government's health education intervention. Key features of the innovative project include: a) weekly health education sessions using participatory learning methods for SBHE; b) use of primary school para-teachers instead of teachers as the facilitators of the health education sessions; and c) the use of mobile phone technology to assist pedagogy and learning, and to motivate both students and teachers. These are discussed in the *method of approach* below. In addition the project will try to realistically follow some basic principles, as outlined in UNICEF's quality standards for life-skills education. They include, but are not limited to: a) life-skills education being need-based; b) learning outcomes being linked to behavioral objectives; c): teaching methods being linked to learning outcomes, are age, and gender relevant, simple, use participatory skills building methods, and support reinforcement; d) training of trainers in teaching methods; e) emphasizing the importance of a safe and protective learning environment. *If proven successful and cost-effective, this project will have implications for the replication of innovative health education to Bangladesh's 20 million SAC, their parents, teachers and communities. As this approach has not been tried elsewhere, it provides the opportunity for adoption by health education projects in other countries, esp. in Asia.*

Objectives

This proposed project aims to evaluate the **impact and cost-effectiveness** of improved innovative school health and nutrition education (ISHNE) interventions at primary schools through different delivery mechanisms on **behavior change, health outcomes and school attendance**. Means of delivery would be through para-teachers trained exclusively for ISHNE with mobile phone technologies to enhance pedagogy, or all the above but with additional targeting of participant children's parents. These means of delivery will be compared with regular HE conducted by primary school teachers. The study will ultimately evaluate the impact on children's health- related knowledge, attitude, practice and behavior (KAPB), their health status, and their school attendance. It will also attempt to evaluate the impact on norm change.

In order to evaluate the impact and cost-effectiveness of ISHNE, the study asks the following ***questions*:**
Question1: Is the proposed ISHNE more effective than current MoPME Health Education program in terms of health-related KAPB improvement and health outcomes?

Question2: Would ISHNE increase people's demand for better health?

Practically, this question asks whether ISHNE alone can motivate participants to adopt healthy behaviors or, whether certain key inputs would accelerate adoption of desired behavioral changes (e.g. provision of soap, micronutrients, etc). Past research has shown that demand for health significantly drops by a small amount of positive price, e.g., people will take up mosquito net if it is given for free, but if a small amount is charged, then suddenly the take up rate drops significantly.[9] We would like to know, if ISHNE can affect the price elasticity of demand for health, and therefore what it means for the provision of services in schools.

Method of Approach

In order to ensure ISHNE is administered smoothly, the project will be under the direct management of a deputy program manager stationed in the field; field activities will be overseen by field officers who will train para-

teachers, who will facilitate health education sessions, and conduct regular monitoring. The project staff will receive continuous support from a Team of Experts and SC staff.

The ***three joint strategies for ISHNE*** are as follows. A key criterion for implementing these three strategies is that they are replicable in terms of method and budget, by the government of Bangladesh and other organizations, to ensure its sustainability and scale-up. Therefore the strategies will adopt low-cost options and options that can be easily integrated in to the existing education system or widespread technologies readily available or already used throughout Bangladesh (e.g. smart phones):

Strategy 1: Weekly health education using participatory methods for SBHE

The proposed project plans to utilize a *weekly timeslot* to conduct health education sessions that will involve participatory SBHE. In the current MoPME proposed Health Education program in Bangladeshi primary schools infrequently infuses heath topics in different subjects into teaching lessons. The proposed ISHNE propose a prescribed weekly time slot, using two of the four physical education class periods prescribed each month for health education. This will permit at least 30 ISHNE sessions per year.

The emphasis of the sessions will be on the development of skills for health using a variety of participatory learning methods (such as open discussions, role-plays, and group work). Skills or life-skills are key determinants to behavior, and it represents the capabilities that allow individuals to demonstrate a behavior and to be better in-charge of the decisions they make. SBHE programs have been found to be effective in promoting healthy behaviors,[10] although rigorous evaluations of this method are few.[11] A participatory SBHE should overcome problems that past school health projects have encountered, such as, not knowing how to use/clean a latrine, or how to organize a cleaning rotation, despite knowing the importance of clean latrine. The materials for sessions will include SC Bangladesh's health education curriculum, which complements the BHBE.

Strategy 2: Use of primary school para-teachers instead of teachers as health education facilitators

The use of para-teacher is based on the practical and incentive-wise concerns that current primary school teachers are too overloaded to take on a new responsibility, while para-teachers, although paid only a fraction of regular teachers, are motivated to teach.[12] Para teachers who are basically new graduates will be provided training on SBHE skills, HE contents and the use of mobile phone technology by the field officer and health education specialist. From para-teachers' perspectives, going through the HE training and practice should prove to be their advantage in improving their capacity and skills as prospective teachers.

Strategy3: Use of mobile phone technology to assist pedagogy, learning, and to motivate students and teachers

The use of mobile phone technology in education is not a new concept in high-income countries. However with the cost of mobile devices dropping and the development of applications specifically for education, there are now new possibilities for these devices as pedagogical tools to assist teachers in education in low-income countries as well. The use of such technology is widespread in Bangladesh, easy, inexpensive and attractive not only to engaging children, but also in helping para-teachers, who may not be proficient in participatory approaches or specific subject matter to conduct the sessions with confidence. Although the effectiveness of this technology in conducting health education is not clear, it is believed that simple, clear but strong multimedia messages with real/realistic shown images to children can get them more engaged and have an impact on their behavior.[13] The proposed project expects para-teachers to use a mobile phone projector[14], while conducting health education sessions. The ISHNE contents will be developed based on existing materials, curriculums and audio-visual contents that are already made available in Bangladesh by SC, UNICEF, and other organizations.

In addition to the above three strategies, we attempt community and parental outreach through the School Management Committee (SMC), since their active participation will be crucial to building and maintain a healthy school environment. SC is currently redeveloping a parental session manual to be used in another project, which we can use. Along with regular parental sessions and sensitization, the proposed project will use other strategies to reach communities, e.g school-based health events involving students' initiatives and outreach, inviting community people for free health exam, plays, etc.

In addition to these strategies, the project will have an intervention of soap distribution in order to identify the impact of goods provision vs. awareness raising.

Intervention Design and Evaluation Methodology

The proposed project applies an intervention-control pre-post evaluation, based on cross-cutting randomization design in order to accurately evaluate the impact of ISHNE intervention. Primary schools in pre-determined sub-districts are randomly chosen as intervention and/or control, where intervention schools will receive ISHNE for a year. A baseline survey will be conducted prior to the project commencement, and the endline survey will be conducted after the project completion. While all children in intervention schools will receive ISHNE, the project

will collect data from a randomly selected group of 40 students per school.

To answer the above-mentioned research questions, schools will be divided into two main groups of about 90 primary schools each. The first group of 90 schools will serve as a control where HE activities as currently provided in classrooms for grades 1-5 will continue as before, plus the schools will receive a first aid kit which is not considered to affect the outcome of the study. In the second group of schools, SC will actively intervene to provide ISHNE for grades 1-5, with parental outreach. Half of the first and second group of schools and their target families will receive an additional intervention of soap provision for a year. Thus, the project design has the following components: **Control** (regular in-class HE); **Intervention 1** (ISHNE); **Cross-cutting Intervention 2** (provision of soap).[15] After a baseline survey (Sep-Nov 2011), all primary schools will be randomly assigned to one of the groups, after being stratified according to the (1) school type (government, and registered non-government[16]) and (2) school health infrastructure (latrine, hand-washing facility), since they may affect the outcome of ISHNE intervention. A pilot intervention will be conducted for 1 week in another location (Nov 2011) to ensure that the feasibility of proposed project and make any adjustment necessary. After one academic year of full Intervention (Jan-Nov 2012), endline survey will be conducted from Jan-Feb 2013, followed by data processing and analysis.

Institutional Review Board approval and ethic review board approval will be guaranteed prior to implementation to assure rigor of evaluation methods and publication in peer review journals. This fits in well with Save the Children's emphasis on evidence-based replicable solutions and justifications for future scale-up.

Sample Size

The total sample size for children is 7,200, where data of 40 pupils are collected from each of 180 schools (half intervention and half control). Sample size calculation is based on expected improvement of 0.15 standardized effect size in child health indicators[17], detected with 80% power and 5% significance level, assuming the school level covariate to explain 2.5% of the variance. A sample size of 34 student per schools will be sufficient, however to account for partial compliance and/or attrition (assumed to be 15%), we are collecting 40 students. The sample size calculation utilized intra-cluster correlation of 0.058, which is derived from SC's baseline survey data in a different area in Bangladesh where SC implements an SHN project, however, this should be readjusted once the baseline survey is done for this study site.

Measurement

The key outcomes that will be measured are:

Primary outcomes:

- Knowledge, attitudes, practices and behavior (KAPB) change of school children: pre-post behavioral written test; pre-post observation (hand washing; latrine status; wearing sandals). It is not easy to measure attitudes, practice and behavior. However, the project will develop a questionnaire which includes objective observations regarding other pupils (e.g., whether s/he has seen someone not washing hands after using a latrine), to provide more accurate information. Tests will be supplemented by observations.

Secondary outcomes:

- Heath improvement: pre-post anthropometric and other health indicators (mainly BMI for age-z score, weight, incidence of diarrhea, pneumonia and scabies, clinical diagnosis of anemia). Some useful indicators to discern health improvement in school children are weight change, incidence of diarrhea and incidence of scabies, although collecting data on diarrhea will need to overcome recall bias. The project plans to distribute a recording calendar or notebook to record the incidence each day prior to the survey. In addition, intervention group will be encouraged to keep students' weight and height measure every month, which not only serve us to check for seasonal variation in weight but also to increase their health awareness.
- School attendance rate: school attendance record and the reasons for absence will be kept by the teachers and para-teachers at each school. The project field facilitator will regularly conduct a random check of the record of each school. Dropout rates and grade-repetition rates and the reasons will also be collected.

Tertiary outcomes:

- Costs-effectiveness of each type of intervention: the cost of each intervention will be estimated and cost-effectiveness will be calculated for each outcome indicators (e.g., $ to increase per day school attendance, $ to increase 1kg of weight, $ to reduce diarrhea incidence by 10%) and compared amongst different project interventions (1, control, 2 and their combinations).

These outcome measures will be also combined into composite scores for each outcome category (index family). In addition, the following variables (data) will be collected for background information and the analysis:

- Household characteristics collected from children's parents (20% of surveyed children): member composition; age, sex, health status, job/school, main-caretaker, education, religion, income, type of house, land area, crops, cattle, credit amount, food intake, latrine availability, safe tubewell availability, soap availability

- School characteristics from the headmaster and observation: school type; number of teachers; class & student characteristics (number, sex ratio); latrines (number, type, status); water availability (facility type, location, arsenic status), soap availability; cleaning arrangement; HE-related activities and SMC status
- Market and health environmental background information: price of health-related supplies (supplements; soaps, sandals; installment of water-sealed latrines); availability and proximity of health clinic and doctor

Data Analysis

Preliminary analysis will be conducted on baseline data to control for significant covariates, such as school sanitary infrastructure, that may affect the project result. The stratification of schools will be done before randomizing them into intervention and control groups. Also, baseline data will be used to recalculate the expected impact level and to adjust for intra-cluster correlation within schools. Using both baseline and endline data sets, the impacts (treatment effects) on each indicator will be estimated, and standard errors properly adjusted for the indicators to form a family index (on health-related KAPB, health improvements and school-attendance). Thus treatment effects on each indicator, the composite indicators as well as the mean standardized treatment effects will be estimated. Standard errors will be adjusted for possible intra-cluster correlations in the impact variables, since randomization is done at the school level. For the impact estimations, Intention to Treat (ITT) will be estimated for an overall project impact as well as to assess the project cost-effectiveness, while Treatment on the Treated (TOT) will be estimated to learn the effect on the children who actually received ISHNE and/or soaps, especially in case of significant partial compliance. Additional regressions will be run for a subgroup of samples to identify possible impact of household characteristics on the outcomes. Each impact indicator will be analyzed for its cost-effectiveness as described in the measurement section above.

Project Timelines (Apr 2011-Mar 2014)

Jul 2011	MoA between Meiji Gakuin University and Save the Children; draft questionnaire; pilot questionnaire and make any amendments
Jul-Dec2011	preparation for the baseline; IHE material compilation & development
Sep-Nov 2011	baseline survey (1.5 month) & data cleaning
Nov-Dec 2011	recruitment and training of project staff on RCT; pilot trial of randomized intervention & finalization of research design; selection of schools and randomization
Jan-Nov 2012	intervention (24 months)
Jan-Feb 2013	endline survey (1.5 month) & data cleaning
Mar 2013-Mar2014	data analysis; report/paper drafting and dissemination

Research Site

The proposed project is expected to take place in sub-district of Jhenaidah, which are target districts of SC's PROTEEVA (Promoting Talent Through Early Education) project funded by USAID. These districts are identified as needy districts, and they provide logistical advantage to the proposed project, having already established Save the Children's offices and local partner NGO relations. Although PROTEEVA project has not commenced SHN interventions for primary schools, given the fact that it has begun preparatory activities of community mobilization including parental ISHNE, this project will exclude those schools that are PROTEEVA supported in order to avoid study contamination.

Anticipated Results and Expected Impacts

The anticipated results of the intervention are:

- Significant (statistically detectable) change in health- related KAPB amongst school children
- Significant change in health amongst school children, in particular, 30% reduction in the incidence of diarrhea (based on expected 0.15 standardized effect size) and 11% improvement in BMI-z score[18]
- Significant change in school attendance and dropout rate amongst school children
- Change in health and health-related KAPB amongst the parents and siblings of the targeted children
- Increased demand for health amongst intervened children and their parents
- Para-teachers who are well trained in SBHE

The anticipated results of the evaluation are:

- Identification of the most impactful, cost-effective and sustainable method of health education in improving children's health
- Dissemination and knowledge sharing of successful ISHNE approach to relevant Government bodies and donor/development agencies for replication and scale-up
- Dissemination and knowledge sharing of successful ISHNE intervention evaluation to academia

The proposed project is hoped to be continued and scaled-up throughout Bangladesh and other countries. If indeed this innovative method to SBHE is more impactful and cost-effective than just traditional methods in low-income settings, it should be promoted in all schools. There are 20 million primary school children in Bangladesh and the

impact will be vast. In the long-run, the project is expected contribute to a human capital development, by enabling children to improve their health status and educational attainments, and thereby to afford a better livelihood. This should contribute to the growth and development of the nation.

Project Risks and Description of Proposed Mitigation

The proposed project will provide school children and a subgroup of their families with needed health education and it is not expected that no particular health or physical risk is associated with the project. A potential risk is because the style of teaching is closer and more interactive – it is important that the adults participating (teachers and others) are aware of and if possible has signed a child protection code of conduct. There may be risk of children withdrawing from the evaluation for varied reasons. If such case happens, the children will be followed up as much as possible. To mitigate these potential risks, children, their parents/guardians and teachers will be informed of the project details prior to the onset of the baseline surveys. Parent-teacher association meetings will be held with the parents and guardians of children enrolled in the participating grades 1-5 to describe the purpose of the surveys, the procedures that will be followed, and the benefits of participation. Information sheets will be provided to the parents or guardians for their review. For those parents who do not attend these meetings, follow-up will be made through community leaders and household visits. Parents or guardians of participating school children will be asked to sign consent forms for their child (if aged under 18 years) to participate in the research study. If a parent, guardian or older child is unable to read or write, his/her fingerprint will be used in place of a signature, and program staff will also obtain a signature from a witness to the informed consent discussion. Parents and guardians will have the chance to ask questions and will be informed that participation of their child in the study is completely voluntary and that they may withdraw from the study at any time. Verbal assent to participate in the study will also be obtained from the child at the time of screening.

There may also be risk of partial compliance amongst children, (para-)teachers and parents. For children, the project will make sure that (para-)teachers take school attendance record every day. For para-teachers who will take the responsibilities of delivering ISHNE will be paid a salary by the project, and it is expected that partial compliance can be largely avoided. For parents, small incentives, such as a bag of lentils, may be given for their participation.

For possible contamination problems, such as Hawthorne and John Henry Effects, i.e., changes in the behavior of the treatment and control group, respectively, due to the fact that evaluation is taking place, the project will try to first identify the degree of interactions between different schools. In case that schools are in vicinity to each other with certain degrees of interactions, the project may clustering the neighboring schools for randomization. Another possible mitigation is through provision of some minor intervention, which would not affect the measurably of our intervention, so that people are not so aware of being the intervention and/or control. However, this option may prove to be practically difficult. Another way to amend these effects are to collect data for some time, as such effects won't last for long, especially after the completion of formal evaluation, but this will be outside the scope of this proposed project. We shall identify the likely magnitude of these effects and possible mitigation measures through initial pilot phase.

All records will be kept confidential and ID numbers will be used to identify the individuals in order to protect private information. No individual identities will be used in any reports or publications resulting from the study.

Where children are identified to suffer from serious illness during the survey and/or implementation phase, s/he will be referred to the nearest health facility for appropriate treatment.

The proposed project will receive Institutional Review Board approval and ethic review board approval prior to implementation to assure rigor of evaluation methods and publication in peer review journals.

Gender Integration

The proposed project will benefit boys and girls equally. The project promotes gender equality by extending its benefits equally to both boys and girls so that they can develop and realize their human rights. By providing key health messages to both boys and girls and instilling important healthy behaviors, the project will ensure their health and well-being as well as those of their families, now and in the future. Girls often serve as the family caretakers who are responsible for siblings and other family members who become ill from all causes. By arming them with basic yet critical life-skills, the project hopes to extend benefits to the wider family and community.

The evaluation of this study will be analyzed and disaggregated by gender to determine if there are any other social or cultural causes that may create additional barriers for improving girls'overall health and educational outcome.

Amount of Effort to be Employed

SC will work in partnership with MoPME, MoHFW, Directorate of Primary Education, Directorate General of Health Services at national, district and sub-district levels, as well as local NGOs to implement the proposed interventions and operations research. It will also have substantial contribution from experienced researchers with different expertise: health and hygiene, HE, economics, biological anthropology. Existing fully- funded SC's staff

will also dedicate time to this project, bringing years of research and program experience from SHN. These include SC's SHN specialist based in India; and the SHN program manager, deputy program manager, and deputy country director based in Bangladesh. The project intervention and evaluation design will be guided by a team of experts that include 2 economists, 2 biological anthropologist, 1 health education specialist, 1 health specialist with extensive experience working in Bangladesh and other developing countries (see bios in the supporting information section). The leading experts are Dr. Makiko Omura (economist),Dr. Jun Kobayashi (medical doctor), Dr. Towa Tachibana (economist) and Dr. Takeshi Sakurai (economist). A health education specialist with significant field and thematic experience will also be consulted during the design of the interventions. For project implementation and monitoring, there will be 6 field officers with regional familiarity, supervised by a Deputy Project Manager on site. There will be additional personnel for finance and administration. The survey team who will conduct baseline and endline survey will be recruited separately from the project staff in order to ensure the neutrality of survey. The project staff will be full-time personnel recruited by SC, and trained by the team of experts for at least one month prior to the project start to ensure that randomized-controlled intervention is strictly observed and to ensure the quality of data. The collected data by the survey team will be re-checked, cleaned and analysed by the team of experts to be published in peer reviewed journals, disseminated to development communities and the government. The project results and process will be rigorously reviewed with SC, and if it is proven successful, a scale-up will be sought in Bangladesh and other countries.

Proposed Duration of the Activity
January 2012 – November 2012 for the ISHNE project implementation
April 2011 – March 2014 for the whole project

ENDNOTES

---

[1] Mobile phone technology refers to technology which is portable, which include mobile phones, smart phones or Android phones which use a variety of communication technologies including wireless, Bluetooth, GSM, GPRS, dial up services, and virtual private networks.

[2] Jukes, Matthew, Drake, Lesley, Bundy, Donald (2008) "School health, nutrition, and education for all: Levelling the playing field" *CABI Publishing*

[3] Luby, Agboatwalla, Feikin, Painter, Billhimer, Altaf and Hoekstra (2005) "Effect of handwashing on child health: a randomized controlled trial" *Lancet* (366).

[4] (1) Madajewicz, Pfaff, van Geen, Graziano, Hussein, Momotaj, Sylvi and Ahsan (2007) "Can information alone change behavior? Response to arsenic contamination of groundwater in Bangladesh," *Journal of Development Economics* (84). (2) Dupas (2011) "Do Teenagers Respond to HIV Risk Information? Evidence from a Field Experiment in Kenya," *American Economic Journal: Applied Economics*, 3 (1).

[5] UNESCO, EFA 2000 Assessment: Thematic Study on School Health and Nutrition, UNESCO, Paris, 2001.
Kirby, D., Laris, B.A., and Rolleri, L., Sex and HIV Education Programs for Youth: Their Impact and Important Characteristics, ETR, Santa Cruz, CA, 2006. Retrieved January 20, 2009 from
<http://www.etr.org/recapp/programs/SexHIVedProgs.pdf>
www.casel.org/sel/impacts.php

[6] (1) Miguel and Kremer (2004) "Worms: Identifying Impacts on Education and Health in the Presence of Treatment Externalities," *Econometrica*, Vol. 72, No. 1. (2) Mascie-Taylor, Karim, Karim, Akhtar, Ahmedd, and Montanari (2003) "The cost-effectiveness of health education in improving knowledge and awareness about intestinal parasites in rural Bangladesh," Economics and Human Biology 1.

[7] Information provided by the Bangladesh Directorate General of Health Services (DGHS, 2010). Note that the official statistics only provide a partial view of actual situation since majority of sick children are not admitted to a hospital. In the baseline survey of Meherpur (2008), less than 3 % of children suffering from diarrhoea actually "visited" a hospital.

[8] Save the Children (2010) Policy Brief. Being Healthy to Learn and Learning to be Healthy.

[9] Cohen, J. and Dupas, P. (2010) “Free Distribution or Cost-sharing? Evidence from a Randomized Malaria Prevention Experiment,” Quarterly Journal of Economis,” Vol 125(1), February.

[10] Shuey, D. A., Babishangire, B. B., Omiat, S., & Bagarukayo, H. (1999). “Increased sexual abstinence among in-school adolescents as a result of school health education in Soroti district, Uganda,” *Health Education Research: Theory and Practice* 14, 411-419.

[11] Mellanby A.R., Rees J. B. & Tripp J. H. (2000) “Peer-led and adult-led school health education: a critical review of available comparative research,” *Health Education Research: Theory and Practice* 15(5), 533-545

[12] The para-teacher strategy is proven to be successful in another Save the Children project currently going on in Meherpur district of Bangladesh. Some schools which are not supported by Save the Children have even started to recruit para-teachers with their own initiative and funds. The expected honorarium of para-teachers in this project is equivalent of what is being paid by these schools, in order to ensure the sustainability of para-teacher system after the project ends.

[13] In Japan, health education films were produced and regularly shown to school children and community members to inform them of the impacts of worms and of the importance of getting rid of them. Some of the key players in the use of technology and mass media for heath include some frontline SMS (http://www.frontlinesms.com/) and Development Media International (http://developmentmedia.net/)

[14] The device can be easily charged in advance when there is power supply, which is recommended due to Bangladesh’s unreliable power supply.

[15] Comparisons will be made amongst the following groups: (1) 1 vs control: to assess the impact of ISHNE compared to control; (2) 1with 2 vs 1 without 2 : to assess the impact of ISHNE alone compared to ISHNE with soap; (3) 1& control with 2 vs 1 & control without 2: to assess the impact of soap provision compared to no provision; (4) 1 with 2 vs control with 2: to assess the impact of ISHNE when soap is provided.

[16] In Bangladesh, the main types of schools are government primary schools, which make up a majority of schools, as well as registered non-government primary schools. These schools differ in the quality of their infrastructure, and resources. Therefore it is important to stratify by school type.

[17] The standardized effect size of 0.15 is a conservative estimate, but considered to be a reasonable degree of health improvement.

[18] As there are no data currently available for child health indicators in the study site, these figures may need to be adjusted after the baseline survey. Current figures are based on the available data of school children in Meherpur, Bangladesh where Save the Children operates a SHN project. For reference, Luby et al. (*op. cit.*) found 53% lower incidence of diarrhoea and 34% lower incidence of impetigo for those under-15year-old children compared to the control in a 1 year randomized controlled intervention of hand-washing message plus provision of soap in Pakistan.
