# Supplementary material for: The effects of skill-based health education—A randomised-controlled intervention in primary schools in rural Bangladesh
Source: PLoS One. 2025 Jul 11;20(7):e0327325. doi: 10.1371/journal.pone.0327325 (PMC12250694; doi:10.1371/journal.pone.0327325)
Supplement: S1 Zip — S1 Fig. Project School Map in Jhenaidah, Bangladesh. S1 Table. Endline (non-DID) estimation of family-wise mean-standardised effect in average effect size on nine outcome families adjusting for baseline covariates (all children). S2 Table. DID estimation of family-wise mean-standardised effect in average effect size on nine outcome families with additional covariates (all children). S3 Table. DID estimation of family-wise mean-standardised cross-cutting HESP-treatment effect in average effect size on five selected outcome families with additional covariates (all children). S4 Table. HE-treatment effects on single outcomes (selected outcomes) (all children; children in both surveys) S1 File. Study Protocol. S1 Checklist. CONSORT Checklist. (ZIP) [file pone.0327325.s001.zip › supplements/S3 Table.pdf]

S3 Table. DID estimation of family-wise mean-standardised cross-cutting HESP-treatment effect in average effect size on five selected outcome families with additional covariates (all children)

	**Primary Outcomes**											
	**(P1) handwashing**				**(P2) dentalcare**				**(P3) overall hygiene**			
	AES-coefficient [95%CI] [p-value]				AES-coefficient [95%CI] [p-value]				AES-coefficient [95%CI] [p-value]			
HE-treatment	0.141*	0.142*	0.142*	0.140*	0.170*	0.169*	0.170*	0.167*	0.163**	0.142**	0.141**	0.139**
	[0.02,0.26]	[0.02,0.26]	[0.02,0.26]	[0.02,0.26]	[0.04,0.30]	[0.04,0.30]	[0.04,0.30]	[0.03,0.30]	[0.04,0.29]	[0.04,0.25]	[0.04,0.25]	[0.03,0.24]
	[0.020]	[0.020]	[0.020]	[0.021]	[0.011]	[0.013]	[0.012]	[0.014]	[0.009]	[0.009]	[0.008]	[0.010]
HESP- treatment	0.201**	0.200**	0.208***	0.199**	0.111+	0.112+	0.122+	0.11	0.179**	0.156**	0.164**	0.154**
	[0.08,0.32]	[0.08,0.32]	[0.09,0.33]	[0.08,0.32]	[-0.02,0.24]	[-0.02,0.24]	[-0.01,0.25]	[-0.02,0.24]	[0.06,0.30]	[0.05,0.26]	[0.06,0.27]	[0.05,0.26]
	[0.001]	[0.001]	[0.001]	[0.001]	[0.095]	[0.096]	[0.066]	[0.103]	[0.003]	[0.003]	[0.002]	[0.003]
SP-treatment	-0.084	-0.083	-0.079	-0.084	-0.047	-0.05	-0.046	-0.051	-0.093	-0.08	-0.077	-0.082
	[-0.20,0.03]	[-0.20,0.03]	[-0.20,0.04]	[-0.20,0.03]	[-0.18,0.08]	[-0.18,0.08]	[-0.18,0.08]	[-0.18,0.08]	[-0.21,0.02]	[-0.18,0.02]	[-0.18,0.02]	[-0.18,0.02]
	[0.162]	[0.168]	[0.183]	[0.161]	[0.482]	[0.457]	[0.483]	[0.449]	[0.109]	[0.125]	[0.132]	[0.116]
HE-group	-0.031	-0.036	-0.024	-0.031	-0.100+	-0.110+	-0.097+	-0.105+	-0.069	-0.062	-0.05	-0.057
	[-0.13,0.06]	[-0.13,0.06]	[-0.11,0.07]	[-0.13,0.06]	[-0.21,0.01]	[-0.22,0.00]	[-0.20,0.00]	[-0.21,0.00]	[-0.16,0.02]	[-0.15,0.03]	[-0.13,0.03]	[-0.14,0.03]
	[0.525]	[0.465]	[0.599]	[0.516]	[0.068]	[0.053]	[0.062]	[0.056]	[0.141]	[0.169]	[0.228]	[0.198]
HESP-group	-0.056	-0.059	-0.052	-0.053	-0.035	-0.048	-0.042	-0.042	-0.054	-0.052	-0.044	-0.045
	[-0.16,0.05]	[-0.16,0.04]	[-0.15,0.04]	[-0.15,0.05]	[-0.14,0.07]	[-0.15,0.06]	[-0.14,0.06]	[-0.14,0.06]	[-0.15,0.04]	[-0.14,0.04]	[-0.13,0.04]	[-0.13,0.04]
	[0.284]	[0.267]	[0.287]	[0.297]	[0.508]	[0.381]	[0.404]	[0.430]	[0.244]	[0.262]	[0.287]	[0.306]
SP-group	0.013	0.008	0.019	0.015	-0.085	-0.096	-0.084	-0.089	-0.01	-0.012	0	-0.004
	[-0.09,0.12]	[-0.10,0.12]	[-0.08,0.12]	[-0.09,0.12]	[-0.20,0.03]	[-0.22,0.02]	[-0.19,0.03]	[-0.21,0.03]	[-0.11,0.09]	[-0.11,0.08]	[-0.09,0.09]	[-0.10,0.09]
	[0.806]	[0.880]	[0.717]	[0.780]	[0.157]	[0.118]	[0.134]	[0.137]	[0.842]	[0.805]	[0.999]	[0.929]
period	0.673***	0.639***	0.649***	0.671***	0.419***	0.338***	0.349***	0.373***	0.570***	0.510***	0.526***	0.548***
	[0.59,0.76]	[0.55,0.73]	[0.56,0.74]	[0.58,0.76]	[0.32,0.52]	[0.24,0.44]	[0.25,0.45]	[0.27,0.47]	[0.48,0.66]	[0.43,0.59]	[0.45,0.60]	[0.47,0.63]

	[0.000]	[0.000]	[0.000]	[0.000]	[0.000]	[0.000]	[0.000]	[0.000]	[0.000]	[0.000]	[0.000]	[0.000]
school type	-0.068**	-0.066*	-0.053*	-0.062*	-0.091**	-0.093**	-0.078**	-0.087**	-0.079**	-0.075**	-0.061**	-0.070**
	[-0.12,-0.02]	[-0.12,-0.01]	[-0.10,-0.01]	[-0.11,-0.01]	[-0.15,-0.03]	[-0.15,-0.03]	[-0.13,-0.02]	[-0.14,-0.03]	[-0.13,-0.03]	[-0.12,-0.03]	[-0.10,-0.02]	[-0.11,-0.03]
	[0.009]	[0.013]	[0.030]	[0.014]	[0.002]	[0.003]	[0.005]	[0.003]	[0.001]	[0.002]	[0.003]	[0.001]
sex	0.089***	0.087***	0.090***	0.086***	0.040**	0.040**	0.043**	0.037**	0.075***	0.073***	0.076***	0.072***
	[0.07,0.11]	[0.07,0.11]	[0.07,0.11]	[0.07,0.10]	[0.01,0.07]	[0.01,0.07]	[0.02,0.07]	[0.01,0.06]	[0.06,0.09]	[0.06,0.09]	[0.06,0.09]	[0.06,0.09]
	[0.000]	[0.000]	[0.000]	[0.000]	[0.002]	[0.002]	[0.001]	[0.005]	[0.000]	[0.000]	[0.000]	[0.000]
child age		0.035***				0.039***				0.041***		
		[0.03,0.04]				[0.03,0.05]				[0.03,0.05]		
		[0.000]				[0.000]				[0.000]		
wealth index			0.101***				0.117***				0.104***	
			[0.09,0.12]				[0.10,0.13]				[0.09,0.12]	
			[0.000]				[0.000]				[0.000]	
parents' literacy				0.056***				0.080***				0.059***
				[0.04,0.07]				[0.06,0.10]				[0.05,0.07]
				[0.000]				[0.000]				[0.000]
N	16181	16170	16171	16181	16183	16172	16173	16183	16163	16152	16153	16163

	**(P6) knowledge**				**(P6E) knowledge + extra (endline)**				**(I1) cold-related symptoms**		
	AES-coefficient [95%CI] [p-value]				AES-coefficient [95%CI] [p-value]				AES-coefficient [95%CI] [p-value]		
HE-treatment	0.397***	0.398***	0.398***	0.396***	0.18***	0.17***	0.18***	0.17***	-0.028	-0.027	-0.027
	[0.24,0.55]	[0.25,0.55]	[0.25,0.55]	[0.24,0.55]	[0.10,0.25]	[0.09,0.24]	[0.11,0.25]	[0.10,0.25]	[-0.07,0.02]	[-0.07,0.02]	[-0.07,0.02]
	[0.000]	[0.000]	[0.000]	[0.000]	[0.000]	[0.000]	[0.000]	[0.000]	[0.250]	[0.258]	[0.255]
HESP- treatment	0.403***	0.404***	0.408***	0.402***	0.16***	0.15***	0.17***	0.16***	-0.031	-0.033	-0.031
	[0.25,0.56]	[0.25,0.56]	[0.25,0.56]	[0.25,0.56]	[0.09,0.23]	[0.08,0.23]	[0.10,0.24]	[0.09,0.23]	[-0.08,0.02]	[-0.08,0.02]	[-0.08,0.02]
	[0.000]	[0.000]	[0.000]	[0.000]	[0.000]	[0.000]	[0.000]	[0.000]	[0.210]	[0.192]	[0.220]

SP-treatment	-0.072	-0.067	-0.069	-0.071	-0.06+	-0.07+	-0.05+	-0.06+	0.034	0.034	0.035
	[-0.22,0.08]	[-0.22,0.08]	[-0.22,0.08]	[-0.22,0.08]	[-0.12,0.01]	[-0.13,0.00]	[-0.11,0.01]	[-0.12,0.01]	[-0.01,0.08]	[-0.01,0.08]	[-0.01,0.08]
	[0.357]	[0.385]	[0.376]	[0.357]	[0.073]	[0.052]	[0.100]	[0.074]	[0.166]	[0.157]	[0.154]
HE-group	-0.046	-0.054	-0.043	-0.047					0.019	0.015	0.017
	[-0.19,0.09]	[-0.19,0.09]	[-0.18,0.09]	[-0.19,0.09]					[-0.01,0.05]	[-0.02,0.05]	[-0.02,0.05]
	[0.515]	[0.455]	[0.541]	[0.510]					[0.269]	[0.379]	[0.315]
HESP-group	-0.082	-0.088	-0.08	-0.08					0.037*	0.034+	0.034+
	[-0.22,0.06]	[-0.23,0.05]	[-0.21,0.06]	[-0.22,0.06]					[0.00,0.07]	[-0.00,0.07]	[-0.00,0.07]
	[0.245]	[0.218]	[0.247]	[0.251]					[0.037]	[0.053]	[0.051]
SP-group	0.025	0.014	0.028	0.026					-0.004	-0.008	-0.007
	[- 0.12,0.16]	[- 0.13,0.16]	[- 0.11,0.17]	[- 0.11,0.16]					[-0.04,0.03]	[-0.04,0.02]	[-0.04,0.03]
	[0.732]	[0.844]	[0.693]	[0.718]					[0.819]	[0.615]	[0.674]
period	0.830***	0.776***	0.817***	0.829***	-0.04	-0.03***	-0.02***	-0.03***	-0.048**	-0.059***	-0.062***
	[0.72,0.94]	[0.67,0.88]	[0.71,0.92]	[0.72,0.93]	[-0.09,0.01]	[-0.09,0.02]	[-0.07,0.03]	[-0.08,0.02]	[-0.08,-0.01]	[-0.09,-0.02]	[-0.10,-0.03]
	[0.000]	[0.000]	[0.000]	[0.000]	[0.166]	[0.224]	[0.367]	[0.196]	[0.007]	[0.001]	[0.000]
school type	-0.025	-0.023	-0.016	-0.021					-0.004	-0.007	-0.005
	[-0.09,0.04]	[-0.09,0.04]	[-0.08,0.04]	[-0.08,0.04]					[-0.02,0.01]	[-0.02,0.01]	[-0.02,0.01]
	[0.429]	[0.477]	[0.594]	[0.501]					[0.624]	[0.443]	[0.556]
sex	0.026*	0.022*	0.026*	0.023*	0.03*	0.02+	0.03*	0.03*	0.003	0.002	0.003
	[0.00,0.05]	[0.00,0.04]	[0.00,0.05]	[0.00,0.04]	[0.00,0.05]	[-0.00,0.05]	[0.00,0.05]	[0.00,0.05]	[-0.01,0.02]	[-0.02,0.02]	[-0.01,0.02]
	[0.022]	[0.047]	[0.020]	[0.038]	[0.018]	[0.064]	[0.019]	[0.027]	[0.725]	[0.855]	[0.761]
child age		0.056***				0.08***			-0.016***		
		[0.05,0.06]				[0.07,0.09]			[-0.02,-0.01]		
		[0.000]				[0.000]			[0.000]		
wealth index			0.059***				0.08***			-0.021***	

			[0.04,0.07]				[0.07,0.10]			[-0.03,-0.01]	
			[0.000]				[0.000]			[0.000]	
parents' literacy				0.039***				0.03***			-0.014**
				[0.02,0.05]				[0.01,0.04]			[-0.02,-0.00]
				[0.000]	8991			[0.001]			[0.008]
N	16183	16172	16173	16183	0.18***	8991	8991	8991	16172	16173	16183

Notes: Each column represents a separate regression on a family of outcomes applying seemingly unrelated regressions (SUR), estimated by a feasible generalised least squares (FGLS) estimator with cluster-robust standard errors (CRSE). AES-coefficient is the mean-standardised average effect size. Analysis is conducted for all children using the difference-in-differences (DID) model, controlling for school type, child sex and additional covariates, namely, *child age*, *wealth index*, and *parent literacy*. *Wealth index* is created through iterated principal factor, reflecting house structure materials, roof materials, number of rooms, latrine structure and materials, possession of electronic appliances, mobile phones and bikes. *Parents' literacy* reflects whether the child mother and father can read. For each outcome family, estimates are provided for all sample children. Selected outcome families are those for which treatment effect was found to be statistically significant in HE-treatment analysis given in S1 Table above; (P1) *handwashing practice*: handwashing frequency in each occasion (before eating, after defecation, after playing), used substances (soap, ash, mud and/or water only), washing with soap in each occasion, wash with running water, correct washing procedure; (P2) *dentalcare practice*: frequency of dentalcare, frequency of using brush/branch, type of materials used; (P3) *overall hygiene practice*: shoes/footwear wearing at school (frequency), shoes/footwear wearing at home (frequency in latrine and in courtyard), + P1 & P2; (P6) *health/hygiene knowledge*: handwashing procedure, breakfast significance; (P6E) *health/hygiene knowledge + extra:* additional knowledge measured only in the endline, i.e., putting water in latrine before defecating, oral rehydration solution (ORS) making, food pyramid. Significance level: + p<0.1, * p<0.05, ** p<0.01, ***p<0.001; 95% confidence intervals and p-value in brackets.
