# Supplementary material for: The effects of skill-based health education—A randomised-controlled intervention in primary schools in rural Bangladesh
Source: PLoS One. 2025 Jul 11;20(7):e0327325. doi: 10.1371/journal.pone.0327325 (PMC12250694; doi:10.1371/journal.pone.0327325)
Supplement: S1 Zip — S1 Fig. Project School Map in Jhenaidah, Bangladesh. S1 Table. Endline (non-DID) estimation of family-wise mean-standardised effect in average effect size on nine outcome families adjusting for baseline covariates (all children). S2 Table. DID estimation of family-wise mean-standardised effect in average effect size on nine outcome families with additional covariates (all children). S3 Table. DID estimation of family-wise mean-standardised cross-cutting HESP-treatment effect in average effect size on five selected outcome families with additional covariates (all children). S4 Table. HE-treatment effects on single outcomes (selected outcomes) (all children; children in both surveys) S1 File. Study Protocol. S1 Checklist. CONSORT Checklist. (ZIP) [file pone.0327325.s001.zip › supplements/S4 Table.pdf]

S4 Table. HE-treatment effects on single outcomes (selected outcomes) (all children; children in both surveys)

	HE-Treatment Effects on Single Outcomes (Selected Outcomes)															
	(1) Pooled		(1E) Endline		(2) FE		(3) DID		(4) ANCOVA		(5) Constrained Base		(6) Logit		(7) Ologit	
**Outcome**	all	*in both*	all	*in both*	all	*in both*	all	*in both*	all	*in both*	all	*in both*	all	*in both*	all	*in both*
handwashing index	0.372**	*0.365***	0.372**	*0.364***	0.518**	0.518**	0.512**	*0.520***	0.392***	*0.371***	0.487***	*0.483****			1.309*	*1.299*
	[0.013]	*[0.015]*	[0.013]	*[0.016]*	[0.033]	[0.033]	[0.024]	*[0.022]*	[0.008]	*[0.012]*	[0.000]	*[0.000]*			[0.098]	*[0.102]*
handwashing index before eating*	0.195**	*0.198***	0.195**	*0.1980***	0.292**	0.292**	0.297***	*0.295***	0.210***	*0.207***	0.279***	*0.272****			1.448*	*1.414**
	[0.013]	*[0.015]*	[0.013]	*[0.016]*	[0.018]	[0.018]	[0.009]	*[0.011]*	[0.006]	*[0.011]*	[0.000]	*[0.000]*			[0.085]	*[0.092]*
handwashing index after defecation*	0.080**	*0.077***	0.080**	*0.077***	0.166**	0.166**	0.147**	*0.158****	0.085**	*0.076***	0.126***	*0.135****			1.391**	*1.420***
	[0.028]	*[0.035]*	[0.027]	*[0.036]*	[0.017]	[0.017]	[0.018]	*[0.009]*	[0.018]	*[0.040]*	[0.000]	*[0.000]*			[0.026]	*[0.012]*
handwashing index after playing*	0.097	*0.0886*	0.097	*0.088*	0.061	0.061	0.0687	*0.0689*	0.094	*0.086*	0.075*	*0.066*	1.068	*1.014*	1.027	*1*
	[0.278]	*[0.324]*	[0.278]	*[0.327]*	[0.626]	[0.626]	[0.552]	*[0.549]*	[0.292]	*[0.335]*	[0.086]	*[0.183]*	[0.799]	*[0.957]*	[0.878]	*[0.999]*
handwashing with soap before eating	0.067**	*0.068***	0.067**	*0.068***	0.114***	0.114***	0.114***	*0.113****	0.070***	*0.069***	0.103***	*0.101****	1.879***	*1.860****		
	[0.012]	*[0.015]*	[0.012]	*[0.015]*	[0.001]	[0.001]	[0.000]	*[0.001]*	[0.010]	*[0.016]*	[0.000]	*[0.000]*	[0.001]	*[0.002]*		
handwashing with soap after defecation	0.032***	*0.033****	0.032***	*0.033****	0.064**	0.064**	0.056**	*0.063****	0.033***	*0.033****	0.047***	*0.053****	1.560***	*1.711****		
	[0.008]	*[0.007]*	[0.008]	*[0.007]*	[0.018]	[0.018]	[0.016]	*[0.008]*	[0.0057]	*[0.008]*	[0.000]	*[0.000]*	[0.006]	*[0.003]*		
handwashing with soap after playing	0.025	*0.034*	0.025	*0.034*	0.062**	0.062**	0.0480**	*0.061***	0.031	*0.038**	0.042***	*0.053****	1.554**	*1.767***		
	[0.251]	*[0.139]*	[0.250]	*[0.140]*	[0.016]	[0.016]	[0.038]	*[0.012]*	[0.160]	*[0.095]*	[0.000]	*[0.000]*	[0.041]	*[0.010]*		
handwashing with running water	0.012	*0.005*	0.012	*0.0054*	0.023	0.023	0.025*	*0.024*	0.017	*0.011*	0.021***	*0.017***	2.559	*9.978***	1.455*	*1.472*
	[0.414]	*[0.703]*	[0.413]	*[0.695]*	[0.154]	[0.154]	[0.091]	*[0.105]*	[0.2404]	*[0.4374]*	[0.0052]	*[0.0395]*	[0.140]	*[0.040]*	[0.084]	*[0.122]*
handwashing procedures	0.774***	*0.821****	0.774***	*0.820****	0.850***	0.850***	0.807***	*0.838****	0.782***	*0.815****	0.801***	*0.843****	2.758***	*2.933****	3.532***	*3.796****
	[0.000]	*[0.000]*	[0.000]	*[0.000]*	[0.000]	[0.000]	[0.000]	*[0.000]*	[0.000]	*[0.000]*	[0.000]	*[0.000]*	[0.003]	*[0.004]*	[0.000]	*[0.000]*
tooth brushing frequency	0.071***	*0.074****	0.071***	*0.074****	0.094***	0.094***	0.091***	*0.0236*	0.069***	*0.065***	0.087***	*0.087****			2.007***	*1.992****
	[0.006]	*[0.008]*	[0.006]	*[0.008]*	[0.005]	[0.005]	[0.002]	*[0.105]*	[0.007]	*[0.018]*	[0.000]	*[0.000]*			[0.007]	*[0.008]*
dental-care index	0.124***	*0.113****	0.124***	*0.113****	0.148***	0.148***	0.151***	*0.087****	0.131***	*0.115****	0.143***	*0.134****	1.524***	*1.493***	1.717***	*1.713****
	[0.000]	*[0.000]*	[0.000]	*[0.000]*	[0.001]	[0.001]	[0.000]	*[0.005]*	[0.000]	*[0.000]*	[0.000]	*[0.000]*	[0.002]	*[0.013]*	[0.000]	*[0.000]*

wearing footwear at school	-0.001	0.001	-0.001	0.001	0.033*	0.033*	0.0238	0.031**	0.003	0.005	0.017**	0.024***	1.2	1.367		
	[0.898]	[0.843]	[0.897]	[0.861]	[0.075]	[0.075]	[0.115]	[0.043]	[0.6685]	[0.3400]	[0.0226]	[0.0046]	[0.409]	[0.184]		
wearing footwear at home	0.105***	0.085**	0.105***	0.084**	0.164**	0.164**	0.155***	0.155***	0.105***	0.075**	0.137***	0.129***	3.032***	2.780**	1.324**	1.352**
	[0.005]	[0.020]	[0.005]	[0.020]	[0.014]	[0.014]	[0.007]	[0.006]	[0.005]	[0.038]	[0.000]	[0.000]	[0.010]	[0.042]	[0.035]	[0.025]
water latrine before defecate [(a)]	0.069*	0.080*	0.069*	0.080*							0.069*	0.07	1.13	1.189*	1.168*	1.196*
	[0.087]	[0.055]	[0.087]	[0.055]							[0.089]	[0.100]	[0.166]	[0.073]	[0.085]	[0.055]
ORS making knowledge[(a)]	0.238***	0.259***	0.238***	0.259***							0.238***	0.252***	1.726***	1.740***	1.713***	1.728***
	[0.000]	[0.000]	[0.000]	[0.000]							[0.000]	[0.000]	[0.000]	[0.000]	[0.000]	[0.000]
breakfast significant	-0.006	-0.004	-0.006	-0.0042	0.037	0.037	0.043*	0.035	0.135***	-0.004	0.026***	0.023**	0.891	0.813		
	[0.173]	[0.189]	[0.171]	[0.182]	[0.172]	[0.172]	[0.079]	[0.136]	[0.000]	[0.1928]	[0.0060]	[0.0450]	[0.648]	[0.506]		
breakfast eaten today	-0.0012	0.003	-0.0012	0.0027	-0.026	-0.026	-0.0302	-0.0251	0.098***	0.004	-0.01	-0.005	0.523**	0.442***	0.906	0.929
	[0.937]	[0.869]	[0.937]	[0.870]	[0.615]	[0.615]	[0.115]	[0.229]	[0.0000]	[0.8328]	[0.4249]	[0.7170]	[0.028]	[0.009]	[0.256]	[0.432]
cleaned school latrine	0.095***	0.109***	0.095***	0.109***	0.194	0.194	0.104***	0.111***	-0.001	0.108***	0.101***	0.113***	1.725***	1.743***		
	[0.000]	[0.000]	[0.000]	[0.000]	[0.307]	[0.307]	[0.000]	[0.000]	[0.930]	[0.000]	[0.000]	[0.000]	[0.000]	[0.000]		
seen other pupils clean school latrine	0.136***	0.118***	0.136***	0.117***	0.109***	0.109***	0.131***	0.222***	0.003	0.116***	0.132***	0.111***	1.833***	1.685***		
	[0.000]	[0.000]	[0.000]	[0.000]	[0.002]	[0.002]	[0.000]	[0.001]	[0.6103]	[0.000]	[0.000]	[0.000]	[0.000]	[0.001]		
swimming frequency	-0.059	-0.042	-0.059	-0.042	-0.024	-0.024	-0.039	-0.026	-0.046	-0.032	-0.043*	-0.026	0.937	0.99	0.912	0.958
	[0.271]	[0.451]	[0.271]	[0.445]	[0.606]	[0.606]	[0.363]	[0.574]	[0.278]	[0.474]	[0.073]	[0.331]	[0.563]	[0.932]	[0.351]	[0.680]
weight z-score (net of clothes)	-0.020	0.016	-0.020	0.016	-0.033*	-0.033*	-0.032*	-0.033*	-0.031	-0.029	-0.032**	-0.030*				
	[0.648]	[0.732]	[0.648]	[0.730]	[0.094]	[0.094]	[0.094]	[0.088]	[0.208]	[0.123]	[0.047]	[0.069]				
cough	-0.014	-0.016	-0.0139	-0.015	-0.02	-0.02	-0.020	-0.020	-0.014	-0.016	-0.017*	-0.017	0.854	0.847		
	[0.200]	[0.176]	[0.197]	[0.179]	[0.313]	[0.313]	[0.191]	[0.190]	[0.178]	[0.163]	[0.088]	[0.137]	[0.216]	[0.212]		
breathing difficulty	-0.006	-0.008*	-0.006	-0.008*	-0.011*	-0.011*	-0.008*	-0.011**	-0.006	-0.007*	-0.006**	-0.008**	0.631	0.522*		
	[0.132]	[0.078]	[0.132]	[0.078]	[0.067]	[0.067]	[0.090]	[0.040]	[0.121]	[0.083]	[0.044]	[0.020]	[0.176]	[0.095]		
fever	-0.005	-0.009	-0.005	-0.009	-0.023**	-0.023**	-0.018**	-0.023**	-0.006	-0.01	-0.01	-0.014*	0.761	0.698**		
	[0.467]	[0.255]	[0.466]	[0.254]	[0.043]	[0.043]	[0.043]	[0.020]	[0.4051]	[0.1945]	[0.1203]	[0.0618]	[0.108]	[0.040]		

cough in 2wks	-0.009	_-0.013_	-0.009	_-0.013_	-0.03	-0.03	-0.034	_-0.029_	-0.01	_-0.012_	-0.023*	_-0.021_	0.86	_0.87_
	[0.593]	_[0.432]_	[0.591]	_[0.441]_	[0.269]	[0.269]	[0.132]	_[0.192]_	[0.563]	_[0.464]_	[0.070]	_[0.137]_	[0.212]	_[0.261]_
breathing difficulty in 2wks	-0.007	_-0.008_	-0.007	_-0.008_	-0.014	-0.014	-0.012	_-0.013_	-0.007	_-0.008_	-0.009*	_-0.010*_	0.68	_0.641_
	[0.372]	_[0.330]_	[0.372]	_[0.331]_	[0.167]	[0.167]	[0.154]	_[0.136]_	[0.359]	_[0.323]_	[0.070]	_[0.072]_	[0.214]	_[0.177]_
sore throat in 2wks	-0.005	_-0.005_	-0.005	_-0.005_	-0.011*	-0.011*	-0.010**	_-0.011**_	-0.005	_-0.006_	-0.007**	_-0.008*_	0.584*	_0.564*_
	[0.196]	_[0.222]_	[0.196]	_[0.222]_	[0.091]	[0.091]	[0.032]	_[0.033]_	[0.157]	_[0.1741]_	[0.047]	_[0.065]_	[0.094]	_[0.086]_
running nose in 2wks	-0.005	_-0.005_	-0.005	_-0.005_	-0.037	-0.037	-0.041**	_-0.037*_	-0.006	_-0.006_	-0.023*	_-0.02_	0.846	_0.86_
	[0.749]	_[0.777]_	[0.749]	_[0.786]_	[0.174]	[0.174]	[0.050]	_[0.093]_	[0.662]	_[0.740]_	[0.070]	_[0.167]_	[0.118]	_[0.181]_
diarrhea in 2wks	-0.007	_-0.021**_	-0.007	_-0.021**_	-0.021	-0.021	-0.0127	_-0.021*_	-0.001	_-0.021**_	-0.008	_-0.021**_	0.899	_0.804*_
	[0.488]	_[0.032]_	[0.487]	_[0.032]_	[0.103]	[0.103]	[0.285]	_[0.082]_	[0.9071]	_[0.0313]_	[0.3032]	_[0.0207]_	[0.348]	_[0.064]_

Note: HE-treatment effects are given for each outcome shown in the first column for separate regression models (1) ~ (6), with significance level * p<0.10, ** p<0.05, *** p<0.01, and p-value in brackets. Models (1) pooled OLS, (1E) endline OLS, (2) child-level fixed effects (FE); (3) DID estimation with multilevel random-effects (RE) , (6) logit and (7) ordered-logit (ologit) apply cluster-robust standard-error (CRSE).; models (4) Analysis of Covariance (ANCOVA) and (5) Constrained Baseline Analysis (CBA) control for intracluster correlation by adjusting for baseline cluster-mean, and (4) *in both* and (5) additionally adjust for individual-mean (for variable available only in endline, only cluster-mean adjustment is applied). Estimates for logit model and ordered-logit (ologit) are estimated in DID form and results shown are exponentiated (odds-ratio) ; logit results are also shown for ordered variables for valid estimates with sufficient 0 observations. The number of observations shown is general number and may vary by specific outcome variables. (a) *ORS making knowledge* is available only in endline. For ANCOVA *all* estimation, the presented results are adjusted for baseline cluster mean as well as individual baseline value.

Model Specification: Estimations are conducted for (1) pooled and (1e) endline only data by OLS estimator, (2) child-level FE and (3) DID estimation with child-level RE using maximum-likelihood (ML) estimator. For binary or ordered response variables, (6) logit and (7) ordered-logit (ologit) estimations by maximum likelihood estimator are also applied. While models (1)~(3), (5) and (6) apply CRSE, as pointed out by Angrist and Pischke (2009), Cameron and Miller (2015), and others, CRSE is not a panacea. McKenzie (2012) points to the fact that with a single baseline and follow-up, it would require twice the sample size in DID to get the same power as obtained with ANCOVA. The benefits and procedures of applying ANCOVA as well as baseline-constrained model are highlighted in a simulation by Hooper et al. (2018). As detailed in McKenzie (2012), instead of DID which attempts to fully control for the non-random differences in baseline that will likely jeopardize predictive power, we apply Analysis of Covariance (ANCOVA) estimation which is reported to be more efficient that DID or post estimator which uses only the endline data [7].

$(1A)\ \ Y_{ijt} = \delta \cdot t + \beta \cdot T_{ijt} + \varphi \cdot \bar{Y}_{j_{t=0}} + \upsilon \cdot Y_{i_{t=0}} + \varepsilon_{ijt},$

This method can adjust for the baseline cluster mean $\bar{Y}_{j_{t=0}}$ or baseline individual value of the outcome variable$Y_{i_{t=0}}$, or both baseline cluster mean and baseline individual value for more precise results (Klar and Darlington 2004; McKenzie 2012; Hooper et al. 2018). As an alternative to ANCOVA, Constrained Baseline Analysis (CBA) is also applied which allows for random effects of cluster as well as individual nested within cluster. CBA can be applied to all pupils at the endline regardless of having their data taken at the baseline [6]. In order to discern possible effects of attrition and replacement, as well as additional class 1 inclusion, we also present the results of estimation over children of closed cohort who were present in both the baseline and endline surveys (*in-both*).
