# Supplementary material for: The effects of skill-based health education—A randomised-controlled intervention in primary schools in rural Bangladesh
Source: PLoS One. 2025 Jul 11;20(7):e0327325. doi: 10.1371/journal.pone.0327325 (PMC12250694; doi:10.1371/journal.pone.0327325)
Supplement: S1 Zip — S1 Fig. Project School Map in Jhenaidah, Bangladesh. S1 Table. Endline (non-DID) estimation of family-wise mean-standardised effect in average effect size on nine outcome families adjusting for baseline covariates (all children). S2 Table. DID estimation of family-wise mean-standardised effect in average effect size on nine outcome families with additional covariates (all children). S3 Table. DID estimation of family-wise mean-standardised cross-cutting HESP-treatment effect in average effect size on five selected outcome families with additional covariates (all children). S4 Table. HE-treatment effects on single outcomes (selected outcomes) (all children; children in both surveys) S1 File. Study Protocol. S1 Checklist. CONSORT Checklist. (ZIP) [file pone.0327325.s001.zip › supplements/S2 Table.pdf]

S2 Table. DID estimation of family-wise mean-standardised effect in average effect size on nine outcome families with additional covariates (all children)

	Primary Outcomes								
	(P1) handwashing			(P2) dentalcare			(P3) overall hygiene		
	AES-coefficient [95%CI] [p-value]			AES-coefficient [95%CI] [p-value]			AES-coefficient [95%CI] [p-value]		
HE-treatment	0.214***	0.216***	0.213***	0.172***	0.176***	0.171***	0.192***	0.194***	0.190***
	[0.13,0.30]	[0.13,0.30]	[0.13,0.30]	[0.08,0.27]	[0.08,0.27]	[0.08,0.27]	[0.12,0.26]	[0.12,0.27]	[0.12,0.26]
	[0.000]	[0.000]	[0.000]	[0.000]	[0.000]	[0.000]	[0.000]	[0.000]	[0.000]
HE-group	-0.052	-0.048	-0.05	-0.032	-0.029	-0.031	-0.052	-0.048+	-0.05
	[-0.12,0.02]	[-0.12,0.02]	[-0.12,0.02]	[-0.11,0.05]	[-0.10,0.04]	[-0.11,0.04]	[-0.11,0.01]	[-0.10,0.01]	[-0.11,0.01]
	[0.153]	[0.162]	[0.155]	[0.419]	[0.425]	[0.422]	[0.101]	[0.096]	[0.101]
period	0.603***	0.614***	0.634***	0.319***	0.331***	0.353***	0.475***	0.492***	0.512***
	[0.54,0.66]	[0.55,0.67]	[0.57,0.69]	[0.25,0.39]	[0.26,0.40]	[0.28,0.42]	[0.42,0.53]	[0.44,0.54]	[0.46,0.56]
	[0.000]	[0.000]	[0.000]	[0.000]	[0.000]	[0.000]	[0.000]	[0.000]	[0.000]
school type	-0.067*	-0.054*	-0.063*	-0.097**	-0.082**	-0.091**	-0.077**	-0.063**	-0.072**
	[-0.12,-0.01]	[-0.10,-0.01]	[-0.11,-0.01]	[-0.16,-0.03]	[-0.14,-0.02]	[-0.15,-0.03]	[-0.12,-0.03]	[-0.10,-0.02]	[-0.12,-0.03]
	[0.012]	[0.029]	[0.014]	[0.003]	[0.005]	[0.003]	[0.001]	[0.003]	[0.001]
sex	0.088***	0.091***	0.087***	0.041**	0.043**	0.038**	0.074***	0.077***	0.073***
	[0.07,0.11]	[0.07,0.11]	[0.07,0.11]	[0.01,0.07]	[0.02,0.07]	[0.01,0.06]	[0.06,0.09]	[0.06,0.09]	[0.06,0.09]
	[0.000]	[0.000]	[0.000]	[0.002]	[0.001]	[0.005]	[0.000]	[0.000]	[0.000]
child age	0.035***			0.039***			0.041***		
	[0.03,0.04]			[0.03,0.05]			[0.03,0.05]		
	[0.000]			[0.000]			[0.000]		
wealth index		0.103***			0.121***			0.106***	
		[0.09,0.12]			[0.10,0.14]			[0.09,0.12]	

		[0.000]			[0.000]			[0.000]	
parents' literacy			0.057***			0.082***			0.060***
			[0.04,0.07]			[0.06,0.10]			[0.05,0.07]
			[0.000]			[0.000]			[0.000]
N	16170	16171	16181	16172	16173	16183	16152	16153	16163
	**(P4) clean hands**			**(P4E) clean hands + ATP (endline)**			**(P5) nutrition**		
	AES-coefficient [95%CI] [p-value]			AES-coefficient [95%CI] [p-value]			AES-coefficient [95%CI] [p-value]		
HE-treatment	-0.009	-0.007	-0.008	0.06	0.07	0.07	-0.043	-0.042	-0.044
	[-0.12,0.10]	[-0.11,0.10]	[-0.11,0.10]	[-0.03,0.16]	[-0.02,0.17]	[-0.03,0.17]	[-0.10,0.01]	[-0.10,0.01]	[-0.10,0.01]
	[0.873]	[0.897]	[0.878]	[0.196]	[0.128]	[0.146]	[0.120]	[0.124]	[0.105]
HE-group	0.031	0.037	0.032				0.054*	0.057**	0.055*
	[-0.05,0.11]	[-0.04,0.11]	[-0.04,0.11]				[0.01,0.10]	[0.01,0.10]	[0.01,0.10]
	[0.431]	[0.332]	[0.400]				[0.014]	[0.008]	[0.010]
period	0.334***	0.374***	0.394***	0	0.03	0.01	-0.028	-0.023	-0.012
	[0.25,0.42]	[0.29,0.46]	[0.31,0.48]	[-0.10,0.10]	[-0.07,0.13]	[-0.08,0.11]	[-0.07,0.01]	[-0.06,0.02]	[-0.05,0.03]
	[0.000]	[0.000]	[0.000]	[0.971]	[0.545]	[0.806]	[0.154]	[0.233]	[0.554]
school type	-0.037	-0.024	-0.035				-0.01	-0.001	-0.007
	[-0.10,0.02]	[-0.08,0.03]	[-0.09,0.02]				[-0.04,0.02]	[-0.03,0.03]	[-0.03,0.02]
	[0.222]	[0.402]	[0.243]				[0.466]	[0.923]	[0.610]
sex	0.144***	0.149***	0.146***	0.07+	0.06	0.06	0.039***	0.041***	0.038***
	[0.12,0.17]	[0.12,0.18]	[0.12,0.17]	[-0.01,0.16]	[-0.02,0.14]	[-0.02,0.14]	[0.02,0.06]	[0.02,0.06]	[0.02,0.06]
	[0.000]	[0.000]	[0.000]	[0.070]	[0.163]	[0.150]	[0.000]	[0.000]	[0.000]
child age	0.065***			0.05***			0.019***		
	[0.06,0.07]			[0.03,0.08]			[0.01,0.03]		
	[0.000]			[0.000]			[0.000]		

wealth index		0.105***			0.07**			0.065***	
		[0.09,0.12]			[0.02,0.11]			[0.05,0.08]	
		[0.000]			[0.002]			[0.000]	
parents' literacy			0.052***			0.03			0.039***
			[0.03,0.07]			[-0.02,0.08]			[0.03,0.05]
			[0.000]			[0.185]			[0.000]
N	16167	16169	16178	867	867	867	16172	16173	16183

	**(P6) knowledge**			**(P6E) knowledge + extra (endline)**		
	AES-coefficient [95%CI] [p-value]			AES-coefficient [95%CI] [p-value]		
HE-treatment	0.437***	0.439***	0.437***	0.19***	0.20***	0.20***
	[0.33,0.55]	[0.33,0.55]	[0.33,0.55]	[0.14,0.25]	[0.15,0.25]	[0.15,0.25]
	[0.000]	[0.000]	[0.000]	[0.000]	[0.000]	[0.000]
HE-group	-0.079	-0.076	-0.077			
	[-0.18,0.02]	[-0.17,0.02]	[-0.18,0.02]			
	[0.127]	[0.131]	[0.128]			
period	0.750***	0.790***	0.801***	-0.03	-0.02	-0.03
	[0.67,0.83]	[0.71,0.87]	[0.72,0.88]	[-0.09,0.02]	[-0.07,0.03]	[-0.08,0.02]
	[0.000]	[0.000]	[0.000]	[0.229]	[0.376]	[0.201]
school type	-0.023	-0.016	-0.021			
	[-0.09,0.04]	[-0.08,0.04]	[-0.08,0.04]			
	[0.480]	[0.600]	[0.506]			
sex	0.022*	0.026*	0.023*	0.02+	0.03**	0.03**
	[0.00,0.04]	[0.00,0.05]	[0.00,0.05]	[-0.00,0.05]	[0.01,0.05]	[0.00,0.05]
	[0.050]	[0.021]	[0.040]	[0.059]	[0.017]	[0.024]
child age	0.056***			0.08***		

	[0.05,0.06]			[0.07,0.09]					
	[0.000]			[0.000]					
wealth index		0.060***			0.08***				
		[0.04,0.07]			[0.07,0.10]				
		[0.000]			[0.000]				
parents' literacy			0.039***			0.03***			
			[0.02,0.06]			[0.01,0.04]			
			[0.000]			[0.001]			
N	16172	16173	16183	8991	8991	8991			
	**Secondary Outcomes**								
	**(I1) cold-related symptoms**			**(I2) other illness**			**(I3) anthropometry**		
	AES-coefficient [95%CI] [p-value]			AES-coefficient [95%CI] [p-value]			AES-coefficient [95%CI] [p-value]		
HE-treatment	-0.046**	-0.047**	-0.046**	0.019	0.017	0.018	-0.021	-0.021	-0.021
	[-0.08,-0.01]	[-0.08,-0.01]	[-0.08,-0.01]	[-0.04,0.08]	[-0.05,0.08]	[-0.04,0.08]	[-0.06,0.02]	[-0.06,0.02]	[-0.06,0.02]
	[0.008]	[0.007]	[0.007]	[0.562]	[0.587]	[0.568]	[0.277]	[0.282]	[0.285]
HE-group	0.030*	0.029*	0.029*	-0.017	-0.018	-0.018	0.012	0.014	0.011
	[0.01,0.05]	[0.01,0.05]	[0.01,0.05]	[-0.08,0.04]	[-0.08,0.04]	[-0.08,0.04]	[-0.05,0.08]	[-0.05,0.08]	[-0.06,0.08]
	[0.014]	[0.017]	[0.015]	[0.574]	[0.571]	[0.569]	[0.702]	[0.698]	[0.761]
period	-0.031*	-0.041***	-0.045***	-0.171***	-0.182***	-0.184**	0.193***	0.119***	0.140***
	[-0.06,-0.01]	[-0.07,-0.02]	[-0.07,-0.02]	[-0.22,-0.13]	[-0.23,-0.14]	[-0.23,-0.14]	[0.16,0.22]	[0.09,0.15]	[0.11,0.17]
	[0.012]	[0.001]	[0.000]	[0.000]	[0.000]	[0.000]	[0.000]	[0.000]	[0.000]
school type	-0.004	-0.006	-0.005	-0.008	-0.009	-0.008	-0.008	0.008	-0.009
	[-0.02,0.01]	[-0.02,0.01]	[-0.02,0.01]	[-0.05,0.03]	[-0.05,0.03]	[-0.05,0.03]	[-0.07,0.05]	[-0.05,0.07]	[-0.07,0.05]
	[0.653]	[0.463]	[0.578]	[0.705]	[0.664]	[0.712]	[0.773]	[0.809]	[0.768]
sex	0.003	0.002	0.003	0.076***	0.075***	0.076***	-0.046**	-0.050**	-0.048**

	[-0.01,0.02]	[-0.02,0.02]	[-0.01,0.02]	[0.06,0.10]	[0.06,0.09]	[0.06,0.10]	[-0.08,-0.01]	[-0.09,-0.01]	[-0.08,-0.01]
	[0.715]	[0.845]	[0.750]	[0.000]	[0.000]	[0.000]	[0.012]	[0.007]	[0.011]
child age	-0.016***			-0.015***			-0.057***		
	[-0.02,-0.01]			[-0.02,-0.01]			[-0.07,-0.04]		
	[0.000]			[0.000]			[0.000]		
wealth index		-0.021***			-0.015**			0.092**	
		[-0.03,-0.01]			[-0.03,-0.00]			[0.07,0.11]	
		[0.000]			[0.005]			[0.000]	
parents' literacy			-0.014**			-0.006			-0.030**
			[-0.02,-0.00]			[-0.02,0.01]			[-0.05,-0.01]
			[0.006]			[0.346]			[0.008]
N	16172	16173	16183	16162	16164	16173	16130	16120	16130

Notes: Each column represents a separate regression on a family of outcomes applying seemingly unrelated regressions (SUR), estimated by a feasible generalised least squares (FGLS) estimator with cluster-robust standard errors (CRSE) except for (I1) *cold-related symptoms*. AES-coefficient is the mean-standardised average effect size. Analysis is conducted for all children using the difference-in-differences (DID) model, controlling for school type, child sex and additional covariates, namely, *child age*, *wealth index*, and *parent literacy*. *Wealth index* is created through iterated principal factor, reflecting house structure materials, roof materials, number of rooms, latrine structure and materials, possession of electronic appliances, mobile phones and bikes. *Parents' literacy* reflects whether the child mother and father can read. For each outcome family, estimates are provided for all sample children and children present *in both* baseline and endline. Each indicator family includes the following variables: (P1) *handwashing practice*: handwashing frequency in each occasion (before eating, after defecation, after playing), used substances (soap, ash, mud and/or water only), washing with soap in each occasion, wash with running water, correct washing procedure; (P2) *dentalcare practice*: frequency of dentalcare, frequency of using brush/branch, type of materials used; (P3) *overall hygiene practice*: shoes/footwear wearing at school (frequency), shoes/footwear wearing at home (frequency in latrine and in courtyard), + P1 & P2; (P4) *clean hand*s: clean hands by observation, trimmed nails, clean nails; (P4E) *clean hand*s + *ATP*: additional hand cleanliness measured by ATP improvement rate (10% of samples); (P5) *nutrition practice*: breakfast habit, breakfast taken in 3 days, food taken in 3 days, ordered by the richness of nutrition score (none; carbohydrate (and fat); carbohydrate and vitamins; vegetable/animal protein and vitamins; vegetable/animal protein and carbohydrate; vegetable protein, animal protein and carbohydrate; protein, carbohydrate and vitamins); (P6) *health/hygiene knowledge*: handwashing procedure, breakfast significance; (P6E) *health/hygiene knowledge* + *extra:* additional knowledge measured only in the endline, i.e., putting water in latrine before defecating, oral rehydration solution (ORS) making, food pyramid; (I1) *cold-related symptoms*: symptoms at present and in the past two-weeks of cough, breathing difficulty, sore throat, fever, running nose, congested nose; (I2) *other illness*: diarrhoea, stomachache, skin disease, fatigue, dizziness, appetite loss in the past two-weeks; (I3) *anthropometry*: height-, weight-, BMI-z-score. Significance level: + $p<0.1$, * $p<0.05$, ** $p<0.01$, *** $p<0.001$; 95% confidence intervals and p-value in brackets.
