# Supplementary material for: The effects of skill-based health education—A randomised-controlled intervention in primary schools in rural Bangladesh
Source: PLoS One. 2025 Jul 11;20(7):e0327325. doi: 10.1371/journal.pone.0327325 (PMC12250694; doi:10.1371/journal.pone.0327325)
Supplement: S1 Zip — S1 Fig. Project School Map in Jhenaidah, Bangladesh. S1 Table. Endline (non-DID) estimation of family-wise mean-standardised effect in average effect size on nine outcome families adjusting for baseline covariates (all children). S2 Table. DID estimation of family-wise mean-standardised effect in average effect size on nine outcome families with additional covariates (all children). S3 Table. DID estimation of family-wise mean-standardised cross-cutting HESP-treatment effect in average effect size on five selected outcome families with additional covariates (all children). S4 Table. HE-treatment effects on single outcomes (selected outcomes) (all children; children in both surveys) S1 File. Study Protocol. S1 Checklist. CONSORT Checklist. (ZIP) [file pone.0327325.s001.zip › supplements/S1 CONSORT_checklist.pdf]

S1 CONSORT Checklist

CONSORT 2010 checklist of information to include when reporting a cluster randomised trial

		Reporting Item	Page Number*
**Title and Abstract**			
Title	#1a	Identification as a randomized trial in the title.	1
Abstract	#1b	Structured summary of trial design, methods, results, and conclusions	1
**Introduction**			
Background and objectives	#2a	Scientific background and explanation of rationale	2-5
Background and objectives	#2b	Specific objectives or hypothesis	2
**Methods**			
Trial design	#3a	Description of trial design (such as parallel, factorial) including allocation ratio.	5-7
Trial design	#3b	Important changes to methods after trial commencement (such as eligibility criteria), with reasons	NA
Participants	#4a	Eligibility criteria for participants	9-11
Participants	#4b	Settings and locations where the data were collected	5-7
Interventions	#5	The experimental and control interventions for each group with sufficient details to allow replication, including how and when they were actually administered	7-9
Outcomes	#6a	Completely defined prespecified primary and secondary outcome measures, including how and when they were assessed	12-13, 14-15, Table 2 & 3
Outcomes	#6b	Any changes to trial outcomes after the trial commenced, with reasons	NA
Sample size	#7a	How sample size was determined.	9-11
Sample size	#7b	When applicable, explanation of any interim analyses and stopping guidelines	NA
Randomization - Sequence generation	#8a	Method used to generate the random allocation sequence.	9-10

Randomization - Sequence generation	#8b	Type of randomization; details of any restriction (such as blocking and block size)	9-10
Randomization - Allocation concealment mechanism	#9	Mechanism used to implement the random allocation sequence (such as sequentially numbered containers), describing any steps taken to conceal the sequence until interventions were assigned	9-10
Randomization - Implementation	#10	Who generated the allocation sequence, who enrolled participants, and who assigned participants to interventions	9-11
Blinding	#11a	participants (schools; children and parents) at the initial treatment allocation; surveyors collecting the data	10, 17
Blinding	#11b	n/a	NA
Statistical methods	#12a	Statistical methods used to compare groups for primary and secondary outcomes	11-16
Statistical methods	#12b	Methods for additional analyses, such as subgroup analyses and adjusted analyses (externality analysis and cost analysis)	35-38, 42-43
**Results**			
Participant flow diagram (strongly recommended)	#13a	For each group, the numbers of participants who were randomly assigned, received intended treatment, and were analysed for the primary outcome	17, Figure 1
Participant flow	#13b	For each group, losses and exclusions after randomization, together with reason	18-19, Figure 1
Recruitment	#14a	Dates defining the periods of recruitment and follow-up	9
Recruitment	#14b	Why the trial ended or was stopped (non-consent and attrition)	18-19, Figure 1
Baseline data	#15	A table showing baseline demographic and clinical characteristics for each group	Table 2 & 3
Numbers analysed	#16	For each group, number of participants (denominator) included in each analysis and whether the analysis was by original assigned groups	Table4-5, 7-8
Outcomes and estimation	#17a	For each primary and secondary outcome, results for each group, and the estimated effect size and its precision (such as 95% confidence interval)	28-35, Table 4&5
Outcomes and estimation	#17b	For binary outcomes, presentation of both absolute and relative effect sizes is recommended	NA

Ancillary analyses	#18	Results of any other analyses performed, including subgroup analyses and adjusted analyses, distinguishing pre-specified from exploratory	35-44
Harms	#19	All important harms or unintended effects in each group (For specific guidance see CONSORT for harms)	7, 45
**Discussion**			
Limitations	#20	Trial limitations, addressing sources of potential bias, imprecision, and, if relevant, multiplicity of analyses	45-46
Generalisability	#21	Generalisability (external validity, applicability) of the trial findings	46
Interpretation	#22	Interpretation consistent with results, balancing benefits and harms, and considering other relevant evidence	44-46
**Other information**			
Registration	#23	Registration number and name of trial registry	1, 7
Protocol	#24	Where the full trial protocol can be accessed, if available	S1 File
Funding	#25	Sources of funding and other support (such as supply of drugs), role of funders	48

* Note: page numbers optional depending on journal requirements
