# Supplementary material for: The effects of skill-based health education—A randomised-controlled intervention in primary schools in rural Bangladesh
Source: PLoS One. 2025 Jul 11;20(7):e0327325. doi: 10.1371/journal.pone.0327325 (PMC12250694; doi:10.1371/journal.pone.0327325)
Supplement: Attachment — Submitted filename: rebuttal letter.pdf [file pone.0327325.s002.pdf]

The Editors
Plos One

1st May 2024

Dear Sir/Madam,

Thank you for the opportunity to submit our manuscript entitled "*The Effects of Skill-Based Health Education: A Randomised-Controlled Intervention in Primary Schools in Rural Bangladesh*" (Manuscript ID: PONE-D-24-17220, which was previously PONE-D-24-03098). Due to the lack of detailed feedback on our initial submission, we have made inquiries for further clarification on the specific concerns noted by the reviewer. However, as detailed feedback was not forthcoming, we have undertaken a comprehensive review of the manuscript to address the general concerns raised during the review process.

Reviewer's Comment 1: Technical Soundness
The reviewer assessed our manuscript as "partly" technically sound without further elaboration. We have meticulously reviewed our methodology and ensured that all experimental procedures are thoroughly described with appropriate controls and statistical rigor to fully support our conclusions.

Reviewer's Comment 3: Data Availability
The response was marked as "No" regarding the availability of all underlying data. Since specific issues were not detailed, we have proactively ensured that all relevant data are now comprehensively available in the Supporting Information, adhering fully to PLOS ONE's data sharing policy.

Reviewer's Comment 5: Age of Data
The reviewer expressed concerns about the age of the data. We have addressed this by further detailing in our manuscript the ongoing relevance of our findings. Specifically, our analysis of behavioural change among schoolchildren, inter-school spillover effects and the application of cost-effective educational techniques in low-income settings remain crucial and applicable to current challenges in the field.

We believe these revisions adequately address the concerns raised in the initial review and enhance the manuscript's quality and scientific merit. We appreciate further guidance on any specific areas requiring additional attention.

Thank you once again for your consideration.

Yours sincerely,

Makiko Omura
Faculty of Economics, Meiji Gakuin University
makiko@eco.meijigakuin.ac.jp
